# Teaching NeuroImages: A rare genetic cause of spastic paraparesis

**DOI:** 10.1212/WNL.0000000000210110

**Published:** 2024-12-02

**Authors:** Shakya Bhattacharjee, Rekha Siripurapu, Andrew Swale, Yanick J Crow, Christopher Kobylecki

**Affiliations:** 1 Department of Neurology, Queen Elizabeth Hospital, University Hospitals Birmingham NHS Foundation Trust, Birmingham, U.K; 2 Department of Neuroradiology, Walton Centre NHS Foundation Trust, Liverpool, U.K; 3 North West Genomic Laboratory Hub (Liverpool), Manchester Centre for Genomic Medicine, Liverpool Women’s Hospital, Crown Street, Liverpool; 4 MRC Human Genetics Unit, Institute of Genetics and Cancer, University of Edinburgh; Edinburgh, U.K; 5 Laboratory of Neurogenetics and Neuroinflammation, Imagine Institute, INSERM UMR1163, Paris, France>; 6 Department of Neurology, Manchester Centre for Clinical Neurosciences, Northern Care Alliance NHS Foundation Trust, Salford, U.K; 7 Division of Neuroscience, Manchester Academic Health Science Centre, University of Manchester, Manchester, U.K

**Keywords:** spastic, paraparesis, leukoencephalopathy, calcification, cyst

A 37-year-old female born in India developed a gradually progressive gait disturbance and bilateral lower limb stiffness beginning at the age of 15 years. There was no relevant family history or consanguinity. Cranial nerve examination was normal. She had symmetrical spastic paraparesis, left ankle clonus and extensor plantar response. She walked with a spastic gait. Magnetic resonance imaging of brain revealed bilateral asymmetric leukoencephalopathy, and areas of low T2 and gradient echo signal in the subcortical white matter, basal ganglia and thalami (Figure). Spinal imaging was normal. Computed tomography confirmed large volume calcification of caudate, putamen, thalamus and subcortical white matter. She was compound heterozygous for n.*5C>T and n.74G>A variants in *SNORD118* consistent with a diagnosis of leukoencephalopathy with intracranial calcification and cysts (LCC), a rare autosomal recessive cerebral microangiopathy.^1^ Seizures and developmental delay are the most common presentations of LCC, but pyramidal and extrapyramidal features may also be seen.^2^

## Figures and Tables

**Figure 1:**
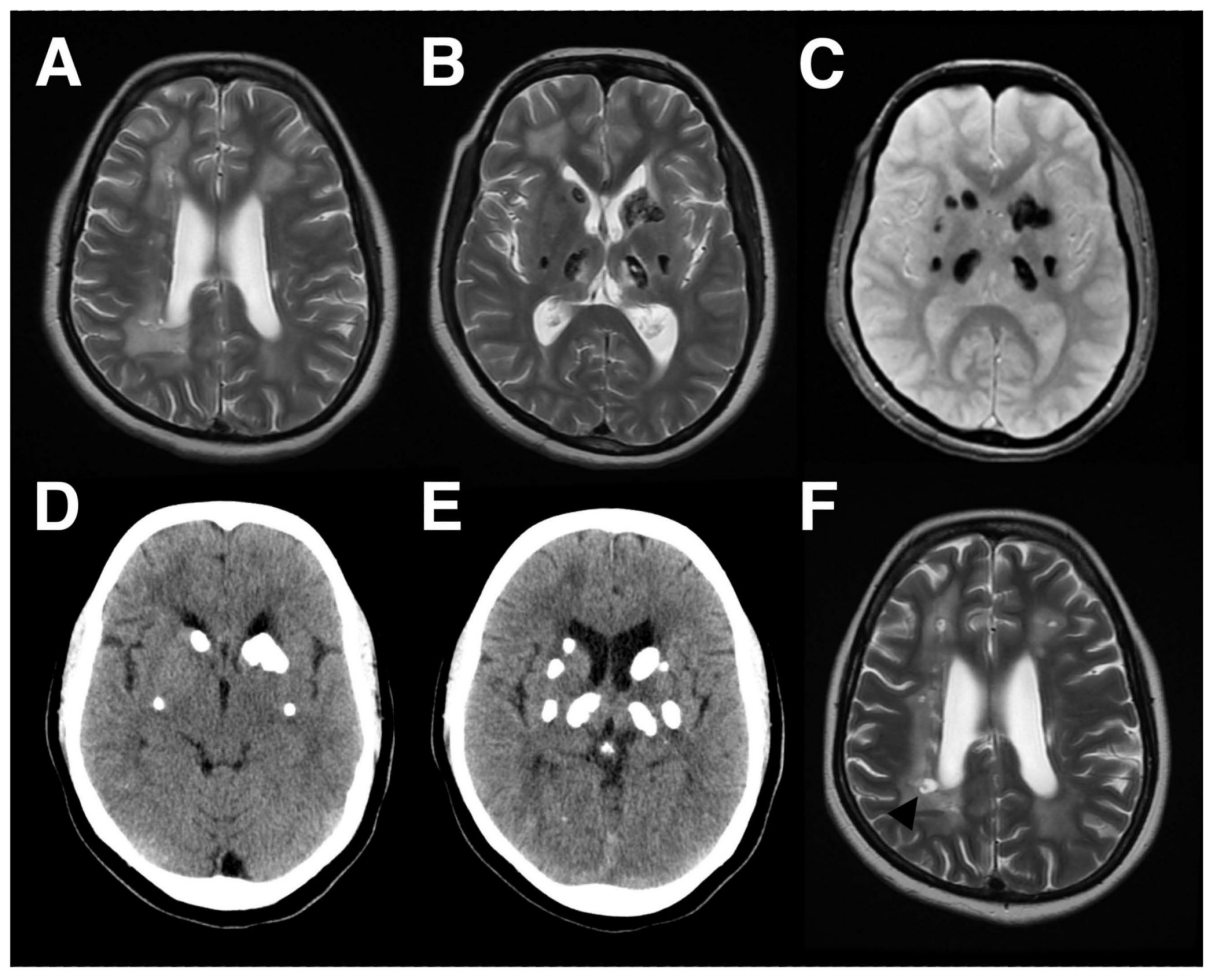
(A,B) Baseline Axial T2 Magnetic resonance (MR) of head shows asymmetric leukoencephalopathy with multifocal low signal areas in basal ganglia and thalami that bloom on Gradient echo (C);(D,E) Computed tomogram confirm corresponding extensive multifocal calcification ; (F) Follow-up axial T2-weighted MR shows development of periventricular cysts (black arrowhead).
